# Factors influencing decision‐making ability of the patient receiving home medical care

**DOI:** 10.1002/jgf2.353

**Published:** 2020-06-25

**Authors:** Akiko Akiyama, Yumi Fukuyama

**Affiliations:** ^1^ Faculty of Health Science Kio University Nara Japan; ^2^ Faculty of Medicine Saga University Saga Japan

**Keywords:** barrier, decision making, end‐of‐life conversation, home care

## Abstract

**Background:**

Patients' decision‐making ability is a substantial barrier to end‐of‐life conversations with doctors. This study aimed to examine factors influencing this ability.

**Methods:**

Altogether, 914 doctors from Japanese home care supporting clinics providing home medical care as of February 2019 participated in this study. Data were collected through an anonymous mailed survey between April and May 2019.

**Results:**

Stepwise multiple linear regression analysis of factors influencing patients’ decision‐making ability revealed the following significant factors: (a) independence level in the daily life of older adults with dementia (B: −0.52), (b) disease name (B: 0.20), and (c) family structure (B: 0.12).

**Conclusions:**

Patients' decision‐making ability regarding conducting end‐of‐life conversations with doctors was characterized; thus, (a) they did not have cognitive impairment, (b) they had cancer, and (c) they lived with a spouse.

## Abstract 2

Patients’ decision‐making ability is a substantial barrier to end‐of‐life conversations with doctors. This study aimed to examine factors influencing this ability.

## 1 INTRODUCTION

Japanese guidelines for end‐of‐life health care and decision processes indicate the importance of providing end‐of‐life care based on the patient's decision‐making ability^1^. Silveira et al^2^ revealed that many patients lacked the ability to make decisions when they needed to and one‐fourth of older patients needed a surrogate for end‐of‐life decision making. This ability is a substantial barrier to end‐of‐life conversations with doctors^3^, ^4^, ^5^; however, predicting who will require surrogate decision making may be difficult^2^. Therefore, this study aimed to examine factors influencing this ability.

## 2 METHODS

### 2.1 Sample

An anonymous survey was mailed to 914 doctors of home care supporting clinics (HCSCs) that were certified by the Japanese Ministry of Health, Labour and Welfare. These doctors were full members of the Japan Network of Home Care Supporting Clinics as of February 2019. This study was performed between April and May 2019; with a cover letter clearly stating the purpose of this study, the right to refuse to participate, strict safeguarding of the data except for the publication of anonymous statistically analyzed data, which does not specify individuals. Informed consent was not required in this study. It was assumed that each subject agreed to join the study of his/her own free will by returning the answered questionnaire. Patients under 10 years old were excluded.

### 2.2 Measurements

We asked respondents to provide information about one recent patient with whom they conducted end‐of‐life conversations. To assess the patient's decision‐making ability, we asked the doctors to assign a score ranging from 1 (highly disagree) to 5 (highly agree) for: “Did the patient have sufficient decision‐making ability?”

### 2.3 Statistical analysis

To analyze the factors influencing this ability, we conducted simple linear regression analysis followed by stepwise multiple linear regression analysis using SPSS 22.0J software for Windows.

## 3 RESULTS

Of the 914 mailed questionnaires, 45 were returned because of incorrect addresses. We received 203 responses, of which one had no answers and was thus excluded (response rate: 23.4%). Of the 202 respondents, two with under 10 years of experience and four with insufficient responses were excluded; 196 were finally analyzed.

Table 1 shows the relationships between decision‐making ability and the doctors' and patients' characteristics. Age, disease name (cancer, senility, heart disease, etc), family structure (with spouse, offspring, etc), daily life independence level of older disabled adults, and daily life independence level of older adults with dementia had *P*‐values ≤.001 in the simple linear regression analysis.

**TABLE 1:** The relationships between decision‐making ability and the characteristics of both respondent doctors and patients

Variables	N, mean ± SD	b	*P*
Characteristics of the respondent doctor			
Age/years	57.6 ± 9.4	−0.061	.394
Gender			
Male	180	0.012	.868
Female	16		
Years of clinical experience	31.3 ± 8.9	−0.061	.397
Years of experience in home medical care	17.6 ± 9.0	−0.027	.709
Characteristics of the patients (n = 196)			
Age/ years	80.3 ± 13.8	−0.420	<.001
Gender			
Male	117	0.137	.055
Female	79		
Disease incidence^a^			
Cancer	109	0.535	<.001
Senility	38	−0.294	<.001
Cerebrovascular	20	−0.214	.003
Heart	15	−0.045	.536
Pneumonia	10	−0.195	.006
Others (diabetes, liver, kidney, PD, others)	Each <10	‐	‐
Family structure			
Alone	19	−0.158	.027
With spouse	57	0.246	.001
With child	76	−0.176	.014
With offspring	24	0.069	.341
Others	20	−0.004	.950
Daily life independence level of the elderly disabled persons^b^			
Not applicable	11	−0.314	<.001
Rank J	4		
Rank A	33		
Rank B	60		
Rank C	84		
Unclear	4		
Daily life independence level of the elderly with dementia^b^			
Not applicable	65	−0.665	<.001
Rank 1	38		
Rank 2	27		
Rank 3	22		
Rank 4	26		
Rank M	14		
Unclear	4		
Patient outcome			
Died at home (Home)	152		
Admitted to the hospital (Hospital)	16		
Placed in the facility (Facility)	25		
Others	3		

Note

Simple linear regression analysis, dependent variables: The patient had sufficient decision‐making ability from 1 (highly disagree) to 5 (highly agree); b: standardized partial regression coefficient;

^a^ Multiple answers allowed; N = 196.

^b^ https://www.mhlw.go.jp/file/06-Seisakujouhou-12300000-Roukenkyoku/0000077382.pdf; cited May 17, 2020. (in Japanese); a is the explanation of Disease incidence.

Table 2 shows the factors influencing this ability. Significant influential factors in stepwise multiple linear regression analysis were daily life independence level of older adults with dementia (B: −0.52), disease name (cancer) (B: 0.20), and family structure (with spouse) (B: 0.12).

**TABLE 2:** Factors influencing decision‐making ability of the patient receiving home medical care

Independent variables	Simple linear regression analysis		Stepwise multiple linear regression analysis	
	B	*P*	b	*P*
Age	−0.420	<.001		
Disease name				
Cancer	0.535	<.001	0.195	.005
Senility	−0.294	<.001		
Family structure				
With spouse	0.246	.001	0.123	.026
Daily life independence level of the elderly disabled persons	−0.314	<.001		
Daily life independence level of the elderly with dementia	−0.665	<.001	−0.520	<.001
F‐value (*P*‐value)			56.95 (<.001)	
Adjusted *R* ^2^			0.472	

Note

Stepwise multiple linear regression analysis, dependent variables: the patient had sufficient decision‐making ability from 1 (highly disagree) to 5 (highly agree); independent variables: variables whose p‐value was less than 0.001 in the simple linear regression analysis; b: standardized partial regression coefficient; R^2:^ determination coefficient; N = 196.

## 4 DISCUSSION

We identified factors influencing patients' decision‐making ability to conduct end‐of‐life conversations with doctors: (a) They did not have cognitive impairment, (b) they had cancer, and (c) they lived with a spouse. Our results correspond to previous research findings showing that patients’ decision‐making ability, specifically cognitive ability, is a substantial barrier to such conversations^2^, ^4^, ^5^, ^6^.

Our results also suggested that family structure differences can affect this ability, that is, their end‐life preferences. Hirakawa et al^7^ indicated that Japanese older adults tended to relinquish their end‐of‐life management, accepting their situation as “fate.” Women were especially eager to maintain good relationships with supporting families. However, contrary to earlier findings^7^, we found that it may be possible to have end‐of‐life conversations based on patients' decision‐making ability because patients who live with their spouse may not need to withhold end‐life preferences.

We identified valuable determining factors influencing patients’ decision‐making ability to conduct end‐of‐life conversations with doctors; however, there were several limitations. First, the response rate was only 23.4%. Second, as we asked the HCSCs to volunteer information about recent patient(s), the result was too few patients for analysis. Third, there were many diseases and conditions with very low incidence, and the numbers were too small to analyze, so the named diseases were limited to only five which involved 10 or more patients (>5% incidence) reported in this study. Fourth, although end of life has a wide meaning as a concept and there are multiple definitions, we did not limit the inclusion (into this study) by a fixed definition of end‐of‐life conversation in this questionnaire. In future studies, we should examine a greater number of patients in this situation.

## 5 CONCLUSION

The characteristics of patients with sufficient decision‐making ability, as assessed by doctors, were as follows: (a) having no cognitive impairment, (b) having cancer, and (c) living with a spouse. For patients who may withhold end‐of‐life preferences, more specific and well‐organized support is necessary.

## CONFLICT OF INTEREST

The authors have stated explicitly that there are no conflicts of interest in connection with this article. This survey protocol was approved by the ethics committee at Kio University, Nara, Japan (approval number: H30‐21).
